# Diet and Nutrition: Temperance in Green Tea

**DOI:** 10.1289/ehp.115-a445a

**Published:** 2007-09

**Authors:** M. Nathaniel Mead

Throughout China and Japan, green tea is considered a staple beverage. Many epidemiologic studies have linked frequent tea intake with a lower incidence of cancer, cardiovascular disease, and neurodegenerative disorders. Consumer interest in the tea’s health benefits has led to the inclusion of green tea extracts in multivitamins and other dietary supplements. But too much of a good thing could prove harmful, according to a review in the April 2007 issue of *Chemical Research in Toxicology* that analyzed the toxic potential of green tea polyphenols.

Currently there are no published epidemiologic studies on the toxicity of green tea supplements. But laboratory research with both rodents and dogs has shown that high doses of the most heavily studied green tea polyphenol, (-)-epigallocatechin-3-gallate (EGCG), cause liver, kidney, and gastrointestinal toxicities.

Case reports on the toxic effects of green tea extracts in humans are also beginning to emerge. “To date, there have been nine anecdotal case reports of liver toxicity in humans associated with consumption of high doses [700–2,000 mg/day] of green tea from dietary supplements,” says lead author Joshua Lambert, an assistant research professor in the Department of Chemical Biology at Rutgers University. “In some cases, the subject stopped taking the supplement and the symptoms resolved, and then the subject started taking the supplement again and liver toxicity returned.” Such observations, albeit anecdotal, suggest that green tea supplements are not without risk.

Cell culture studies have shown that EGCG can cause oxidative stress, although these data now need to be confirmed in animal models. The Rutgers team speculates that some susceptible individuals may carry a particular polymorphism of the gene that codes catecholamine-*o*-methyltransferase, an enzyme critical to the protection of cells against EGCG-mediated oxidative stress and hepatotoxicity. About a quarter of the population have a polymorphism that is associated with low activity of this enzyme. “This is just a hypothesis that we are testing,” says coauthor Chung S. Yang, a chemical biology professor at Rutgers.

Toxic effects tend to arise when people take green tea supplements, which can contain more than 50 times as much polyphenol as a single cup of tea. “People who take less than 500 mg [of green tea concentrate or preparation] per day and spread the dose out over the course of the day are unlikely to have toxic side effects,” says Yang.

Yang adds that some Japanese publications report beneficial effects for the consumption of 10 cups of green tea a day with no apparent harmful effects. At most, people may experience stomach irritation after drinking strongly brewed green tea on an empty stomach. Commercial preparations such as the bottled green teas found in the United States and green tea–flavored gum, bread, candy, ice cream, and desserts found in Asia have very low levels of polyphenols.

At the present time there is no established upper tolerable limit for green tea consumption. The Rutgers review points to the need for epidemiologic studies to test the potential concerns of taking green tea supplements at 500-mg doses or higher. Yang and Lambert hypothesize that people with oxidative stress–related liver diseases such as hepatitis or cirrhosis may be at greater risk of toxic side effects from ingesting high doses of green tea polyphenols. “When a person’s liver is already under stress, toxic effects tend to become amplified,” Yang says. Conversely, he notes there are data showing that low or moderate amounts of green tea have a protective effect against both toxicity and carcinogenesis in target organs—once again supporting the adage “everything in moderation, nothing to excess.”

## Figures and Tables

**Figure:**
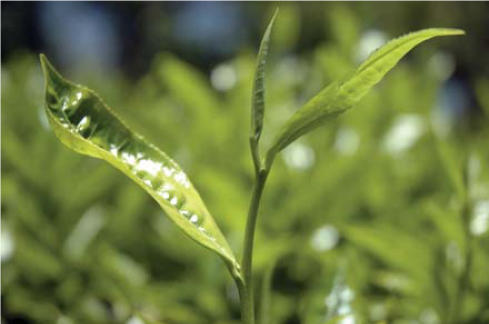
Did you know? Field workers pluck only two leaves and a bud (shown above) during the thrice-yearly tea harvest. To make green tea, leaves of the *Camellia sinensis* plant are specially processed to prevent oxidation. Black, oolong, and white teas are made from the same plant, just using different processes.

